# Large cell anaplastic medulloblastoma metastatic to the scalp: tumor and derived stem-like cells features

**DOI:** 10.1186/1471-2407-14-262

**Published:** 2014-04-16

**Authors:** Angela Mastronuzzi, Evelina Miele, Agnese Po, Manila Antonelli, Francesca Romana Buttarelli, Giovanna Stefania Colafati, Francesca del Bufalo, Roberta Faedda, Gian Paolo Spinelli, Andrea Carai, Felice Giangaspero, Alberto Gulino, Franco Locatelli, Elisabetta Ferretti

**Affiliations:** 1 Department of Hematology/Oncology and Stem Cell Transplantation, Bambino Gesù Children’s Hospital, IRCCS, Piazza Sant’Onofrio 4, 00165 Rome, Italy; 2 Department of Molecular Medicine, Sapienza University, Viale Regina Elena 291, 00161 Rome, Italy; 3 Center for Life NanoScience@Sapienza, Istituto Italiano di Tecnologia, Viale Regina Elena 291, 00161 Rome, Italy; 4 Department of Radiological, Oncological and Pathological Science, Sapienza University, Viale Regina Elena 291, 00161 Rome, Italy; 5 Department of Radiology, Unit of Neuroradiology, Bambino Gesù Children’s Hospital, IRCCS, Piazza Sant’ Onofrio 4, 00165 Rome, Italy; 6 Department of Medico-Surgical Sciences and Biotechnologies, Sapienza University, UOC Oncology Aprilia, Via Giustiniano, 04011, Aprilia, LT, Italy; 7 Department of Neuroscience and Neurorehabilitation, Neurosurgery Unit, Bambino Gesù Children’s Hospital, IRCCS, Piazza Sant’ Onofrio 4, 00165 Rome, Italy; 8 Neuromed Institute, IRCCS, Via Atinense 18, 86077 Pozzilli, IS, Italy

**Keywords:** Medulloblastoma, Stem-like cells, Molecular features, Subcutaneous metastasis

## Abstract

**Background:**

Extraneural metastases (ENM) rarely occur in medulloblastoma (MBL) patients and only few cases of subcutaneous localizations have been described. ENM indicate an aggressive disease associated with a worse prognosis. The characterization of metastatic tumours might be useful to understand their pathogenesis and to identify the most appropriate therapeutic strategies.

**Case presentation:**

We present the case of a child with Large Cell Anaplastic (LC/A) MBL, who developed multiple subcutaneous metastases in the scalp area after a ventriculo-peritoneal shunting procedure. The disease rapidly progressed and the child died despite chemotherapy and primary tumour surgical debulking.

We molecularly classified the tumour as a group 3 MBL; in addition, we derived stem-like cells (SLC) from a metastatic lesion. Primary tumour, metastases and SLC were further analysed, particularly focusing on features linked to the cutaneous dissemination. Indeed, molecules involved in angiogenesis, cell invasion and epidermal growth factor signalling resulted highly expressed.

**Conclusions:**

The present report describes a very rare case of subcutaneous metastatic MBL. The tumour, metastases and SLC have been clinically, pathologically and molecularly characterized. Our case is an example of multidisciplinary approach aiming to characterize MBL aggressive behaviour.

## Background

MBL is the most common malignant brain tumour of childhood, accounting for approximately 15-20% of central nervous system (CNS) malignancies [1]. Despite multimodal therapy, 20% to 30% of MBL recur [2]. Large cell/anaplastic (LC/A) variant is most commonly associated with metastatic disease [3]. Brain and spine secondary localizations are frequent, while extraneural metastases (ENM) are rare (<10% of cases) and occur mostly in bone, lymph nodes, lung and liver [4]. Subcutaneous ENM have been described in very few cases [5,6].

In recent years, high-throughput studies [7-14] allowed to classify MBL into four subgroups (WNT, SHH-Sonic Hedgehog, Group 3 and Group 4) and revealed the existence of gene mutations and of expression patterns linked to a worse prognosis.

Here we report the case of a child affected by LC/A MBL who developed subcutaneous metastases after a ventriculo-peritoneal shunting procedure. We performed histological and molecular analysis to characterize in details this aggressive tumour, particularly focusing on the stem-like cell population.

### Methods

Histology: Paraffin-embedded 3-μm-thick sections from MBL tumour sample were stained with haematoxylin and eosin (H&E). Histology was reviewed by 2 neuropathologists (F.G. and M.A.) and diagnosis was centralized to minimize inter-observer variability.

Immunohistochemistry (IHC): Monoclonal antibody to p53 (DO-7; Dako, Carpinteria, CA, USA; dilution 1:300), and polyclonal anti-Beta catenin antibody (BD Transduction Laboratories, San Jose, CA, USA; dilution 1:100) were used.

Fluorescence in situ hybridization (FISH): Tumour tissue sections were deparaffinised and pre-treated with pepsin before hybridization with c-myc and control (centromere of chromosome 8) probes (Abnova Corporation, Taipei, Taiwan).

Stem-like cells (SLC) culture: Cells were isolated as previously reported [15]. Briefly, fresh tumour was dissociated to single cell suspension and cultured in DMEM/F12 medium supplemented with 0.6% glucose, 25 mg/ml insulin, 60 mg/ml N-acetyl-L-cystein, 2 mg/ml heparin, 20 ng/ml EGF, 20 ng/ml bFGF, 1× penicillin-streptomycin and B27 supplement without vitamin A.

Immunofluorescence: Immunofluerescence was performed as previously described [15]. Primary antibodies used were anti-Sox2 (MAB4343 Millipore) and anti-Nestin (ab6142; Abcam).

RNA extraction, reverse transcription and gene expression analysis: RNA extraction, reverse transcription and gene expression analysis were performed as previously described [16]. RNA from normal cerebella were purchased from Life Technologies. TaqMan Low Density Array was custom designed with TaqMan assays for genes of interest [17,18]. 1 μg RNA was reverse transcribed using High capacity cDNA reverse transcription Kit (Ambion- Life Technologies Corporation, Carlsbad, CA, USA). Gene expression analysis on samples was performed employing an ABI Prism 7900 HT sequence detection system (Applied Biosystems- Life Technologies Corporation, Carlsbad, CA, USA) according to manufacturer’s instructions. Transcripts quantification was expressed in arbitrary units as the ratio of the sample quantity to the calibrator or to the mean values of control samples. All values were normalized to the 4 endogenous gene controls: GAPDH, ß- ACTIN, ß2-MICROGLOBULIN and HPRT. Heat maps were generated employing SpotFire software according to Delta Ct values, as previously described [19].

Cytofluorimetry: Samples were dissociated into single cells and incubated with APC-conjugated anti-CD133 or with isotype control (Miltenyi Biotec, Bergisch-Gladbach, Germany) according to manufacturer’s instructions.

Ethics Committee of Bambino Gesù Children Hospital approved the case study.

## Case presentation

### Patient’s history and clinical features

**Figure 1:**
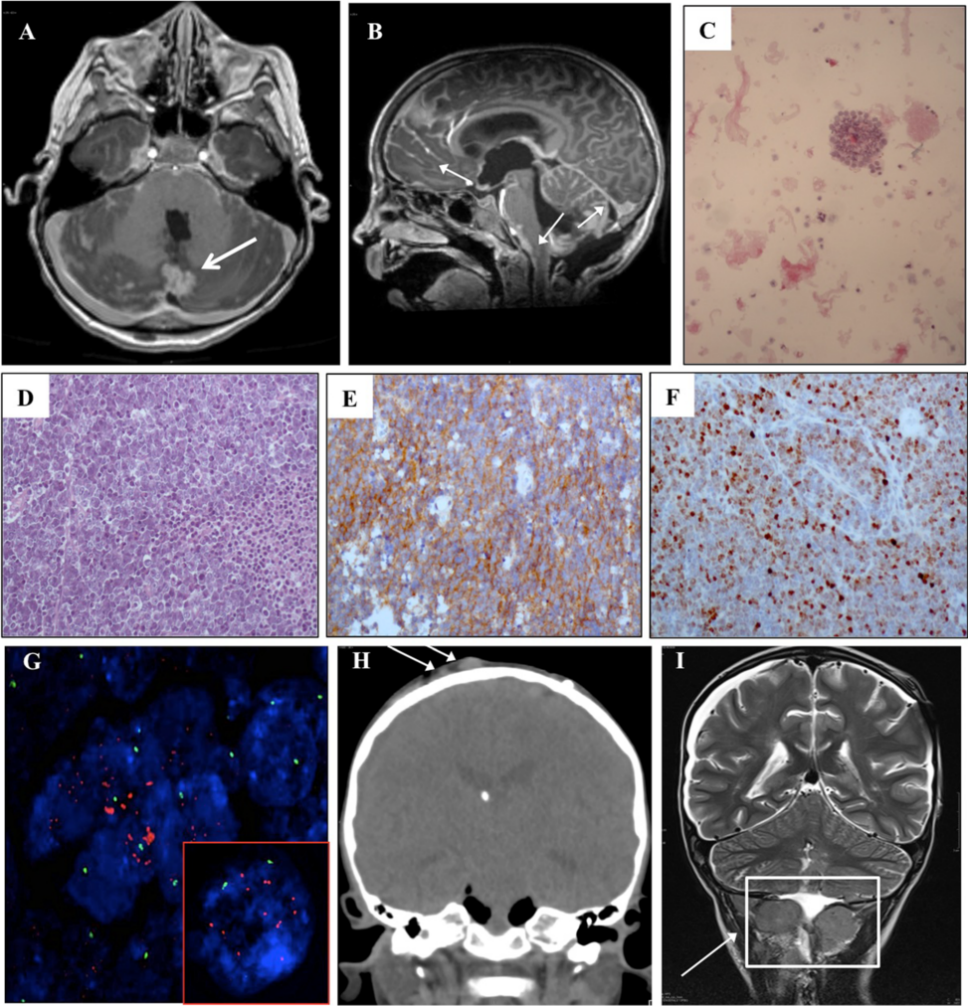
**Radiological and histological features of MBL. (A)** Post-gadolinium axial MR images show pseudonodular area of contrast-enhancement consistent with cerebellar neoplastic residual (arrow). **(B)** Post-gadolinium sagittal MR images show abnormal leptomeningeal enhancement (arrows) along the ventral surface of the mid-brain, cerebellar fissures and cerebral sulci due to leptomeningeal carcinomatosis. **(C)** Cerebrospinal fluid cytology shows the presence of large sized neoplastic cells that appear either singly or in clusters with rosette formation. **(D)** Hematoxylin&eosin staining of the primary lesion showing the presence of neoplastic cells large in size with marked anaplasia and large nuclei with evident nucleoli. Cell wrapping and necrotic phenaomena are also present (arrows). **(E)** Beta-Catenin immunohistochemical evaluation showing membrane and cytoplasmic positivity for beta-catenin with a negative nuclear staining. **(F)** p53 protein is overexpressed by neoplastic cells. **(G)** c-myc oncogene amplification (red spots) detected in neoplastic nuclei (blue) and centromere 8 signals (green spots) using CEP8/BAC as FISH probes. The white box highlights a nucleus in detail where 10 red spots are coupled with two green spots. **(H)** Coronal reformatted CT scan shows two subcutaneous metastases (arrows) with intact underlying calvarial bone. **(I)** Coronal T2-weighted MR image shows voluminous extracranial metastases on the edge of the collection at the site of the former sub-occipital craniotomy, with fascial and muscular infiltration.

A 5 years old male with a recent history of MBL was referred to Hematology/Oncology Department of the Bambino Gesù Children Hospital for obstructive hydrocephalus. A partial resection of MBL had been performed at another institution 20 days earlier. After surgery, MRI revealed a surgical residue associated with a diffuse supra- and infratentorial meningeal involvement (Figure 1A and B) and associated hydrocephalus. Shortly after admission, an endoscopic third ventriculostomy was performed and converted to a ventriculo-peritoneal shunting procedure for intra-operative absence of significant cerebrospinal fluid (CSF) flow through the stoma. CSF resulted positive for neoplastic cells (Figure 1C). The histological revision of the primary lesion confirmed the diagnosis of MBL, variant LC/A (Figure 1D). The tumour resulted negative for nuclear Beta-Catenin (Figure 1E) and positive for p53 staining (Figure 1F). Moreover, c-myc amplification was detectable by fluorescent in situ hybridization (Figure 1G). Systemic chemotherapy based on carboplatin, etoposide and ifosfamide associated with intrathecal topotecan was immediately started and autologous hematopoietic stem cells were harvested after the first cycle. Clinical improvement with recovery of consciousness and disappearance of neoplastic cells in CSF was documented after the first 2 cycles. After the third cycle, the child’s clinical conditions rapidly worsened with progressive neurologic deterioration and occurrence of multiple solid woody masses in the scalp and posterior neck areas (Figure 1H). Surgical debulking was performed, revealing a diffuse infiltration of muscular, fascial and subcutaneous layers of the posterior neck (Figure 1I) and histological examination confirmed the primary diagnosis. The child rapidly progressed and died few days after surgery.

### Molecular features of tumour, metastases and stem-like cells

**Figure 2:**
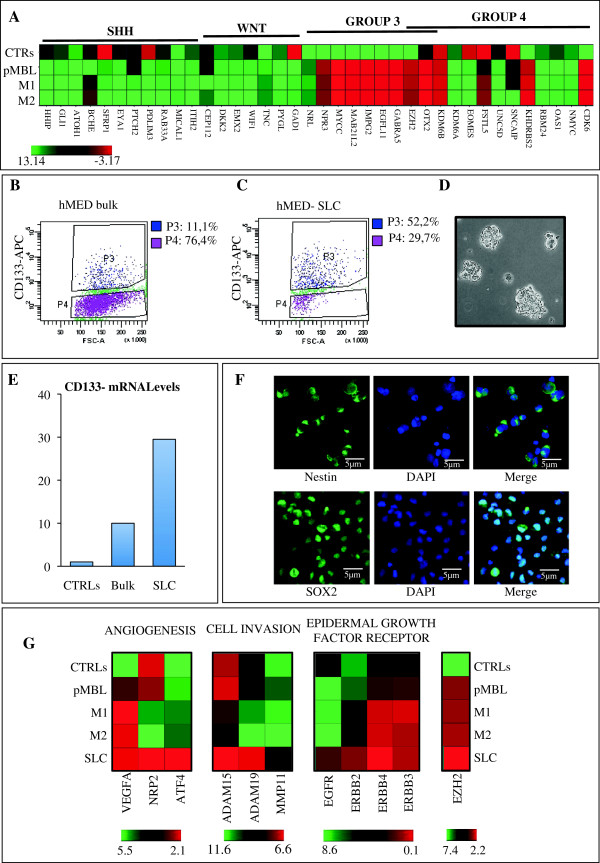
**Molecular characteristics of MBL and derived stem like cells. (A)** Heat map showing mRNA levels of the indicated genes in primary MBL (pMBL), scalp (M1) and neck (M2) metastases compared to normal cerebella (average of n = 8) as control (CTRLs). Genes are grouped depending on the molecular subgroups, which they identify (SHH, WNT, GROUP-3, GROUP-4). A green-red colour scale depicts normalized Delta Ct values (green, lower expression, red, higher expression). **(B-C)** Flow cytometry analysis (FACS) of CD133 in the starting population from neck metastasis (hMED bulk) **(B)** and after 14 days of culture **(C)** (isotypic control not shown). P3 shows percentage of positive cells while P4 shows percentage of negative cells. **(D)** Representative bright field image of neurospheres derived from neck metastasis after 14 days of culture. **(E)** Histograms showing mRNA levels of CD133 in MBL-neck metastasis (bulk) and in its derived SLC compared to normal cerebella as control (CTRLs). **(F)** Immunofluorescence staining with anti-Nestin and anti-Sox2 (green), Hoechst staining (blue) and merge of SLC. Scale bar = 5 μm for all panels. **(G)** Heat maps of expression levels of the indicated genes belonging to the highlighted categories in: pMBL, metastases (M1 and M2), in SLC derived from M2 and in normal adult cerebella as control (CTRLs). A green-red colour scale depicts normalized Delta Ct values (green, lower expression, red, higher expression).

RNA was extracted and retro-transcribed from fresh primary tumour and subcutaneous metastases (neck and scalp). cDNA was analysed on a custom microfluid card with a panel of genes specific for MBL molecular subgroups [17,18]. Over-expression of c-MYC, NRP3, NRL, GABRA5, IMPG2, MAB21L2 and OTX2 was suggestive of a molecular Group 3 for all samples analysed (Figure 2A). To shed light on the potential molecular mechanisms involved in the aggressive behaviour of MBL here described, we isolated its stem cell component (SLC) from neck metastasis. Bulk tumour population harboured CD133 positive cells (11%) (Figure 2B) which were enriched up to 52% in the derived neurospheres (Figure 2D-E). The stem like nature of these cells was further supported by Nestin and SOX2 protein expression (Figure 2F).

We investigated molecular features linked to angiogenesis, cell invasion and epidermal growth factor receptors on the primary tumour (pMBL), metastases (M1 and M2) and SLC (Figure 2G). Most of the analysed genes were highly expressed in the primary lesion versus CTRLs; M1 and M2 had very similar gene expression patterns with some differences (e.g., NRP2 and ADAMs) with respect to pMBL. Interestingly, a higher expression of all analysed genes was detected in SLC (Figure 2G) compared to pMBL and metastases. Of note, high expression of both ERBB2 and ERBB4, among epidermal growth factor receptors, could have a role in the observed subcutaneous seeding (Figure 2G).

Enhancer of zeste homolog 2 (EZH2) was also evaluated due to its reported correlation with aggressive MBL subgroups [20]. Indeed, it resulted overexpressed in all tumour samples as well as in SLC (Figure 2G) when compared to controls.

## Conclusions

MBL is a heterogeneous disease with survival rates ranging from 85% for average risk patients to lower rates (<65%) in the presence of risk factors [21,22]. The identification of MBL histological variant is critical for patient stratification: the presence of desmoplastic or of extensive nodular histology is a strong predictor for low risk disease in early childhood, while LC/A MBL has been associated with high risk and lower survival rates [3]. Indeed, LC/A MBL has been defined as a separate entity in the current WHO classification of CNS tumours [23].

Recently, Von Hoff suggested a better outcome for children with severe anaplastic histology without additional clinical and molecular risk factors (e.g. c-myc amplification, large cell histology, young age and metastases) [3]. Amplification of the proto-oncogene c-myc, a well-known negative prognostic marker for MBL, has been associated to LC/A variant and could contribute to its aggressive behaviour [24,25]. Indeed, the frequency of c-myc amplification has been reported in about 5% of MBL in mixed cohorts [26], while a higher percentage has been reported in LC/A MBL [27]. Among the prognostic markers investigated to predict disease recurrence, only four have been confirmed as related to a poor outcome in a reproducible manner: p53, survivin, erbB-2 expression and amplification of myc genes [2]. In detail, p53 immunopositivity is an adverse prognostic marker [2,28] and somatic TP53 mutations are associated with chemo- and radiotherapy resistance [2,29]. A recent study showed that somatic TP53 mutations are enriched almost exclusively among SHH and WNT subgroup and are highly predictive of extremely poor survival in SHH MBL [30]. Presence of gross anaplasia and c-erbB-2 overexpression status are the most important predictors of recurrence rates [31].

Our patient showed adverse prognostic markers: severe anaplastic histology, c-myc amplification and p53 immunoreactivity. According to the subgroup classification [32], our patient was diagnosed as a group 3 (both for the primary tumour and the metastatic lesions), as expected from LC/A histology with c-myc amplification and with negative nuclear staining for Beta-Catenin. These histopathological features and biological properties might explain the occurrence of secondary localizations, including the early developed ENM.

Subcutaneous metastases have been reported in very few cases [5,6]. Our clinical report is the first in a well-documented LC/A MBL. Differently from what previously described, our patient showed subcutaneous infiltration in the posterior neck region, far from the path of the ventriculo-peritoneal shunt. Moreover, in the final stages of the disease, a cobblestone appearance of the scalp suggested further dissemination of tumour.

Presence of VP shunt has been suggested as a risk factor for MBL metastases [33]; however, the general concept that systemic metastases are more frequent in patients with CSF shunts is not fully accepted [34].

Interestingly, stem cell signatures have been associated with a poor prognosis in tumours, raising the concept that the stem cell population may indeed contribute to the aggressive behaviour [35,36]. Very recently, it has been reported that clonal genetic events observed in metastases can be demonstrated in a restricted sub-clone of the primary tumour, suggesting that only rare cells have the ability to metastasize [37].

The opportunity to compare primary tumour with its metastases and SLC allowed us to highlight novel molecular findings. Our results are in agreement with the disease model based on different compartments: metastases were very similar among each other, while showing some differences versus the primary MBL. Differences were more evident between tumour samples and stem like cells.

Neoangiogenesis and cell invasion molecules were highly expressed in tumour bulk and even more in SLC. The same pattern was observed for the expression levels of molecules involved in EGF signalling, that may be linked to the occurrence of subcutaneous seeding.

In the era of molecular characterization of tumours, the identification of biological mechanisms of aggressiveness might well contribute to develop the most appropriate therapeutic strategies. In the present clinical case, we have shown that both primary tumour cells and SLC express ERBB family members, supporting a possible use of anti-ERBB specific therapies, which are already available [38,39]. In addition, our case expressed EZH2, a molecule that has been recently reported as a critical regulator in MBL growth, thus representing a novel potential therapeutic target [40].

In conclusion, we report a rare case of subcutaneous metastatic LC/A MBL, which was analysed in details through a multidisciplinary approach. The molecular characterization of these aggressive tumours might improve the understanding of their pathogenesis and provide the rationale for targeted therapeutic strategies.

### Consent

Written informed consent was obtained from the patient’s parents for publication of this Case report and of any accompanying images.

## Competing interests

The authors declare that they have no competing interests.

## Authors’ contributions

EM, AP, GPS, RF, AG and EF carried out molecular studies. AM, FdB, GSC, AC and FL clinically followed the patient. MA, FRB, FG carried out pathologic assessments. All authors read and approved the final manuscript.

## Pre-publication history

The pre-publication history for this paper can be accessed here:

http://www.biomedcentral.com/1471-2407/14/262/prepub
